# Activation of PAC1 Receptors in Rat Cerebellar Granule Cells Stimulates Both Calcium Mobilization from Intracellular Stores and Calcium Influx through N-Type Calcium Channels

**DOI:** 10.3389/fendo.2013.00056

**Published:** 2013-05-10

**Authors:** Magali Basille-Dugay, Hubert Vaudry, Alain Fournier, Bruno Gonzalez, David Vaudry

**Affiliations:** ^1^INSERM U982, Laboratory of Neuronal and Neuroendocrine Differentiation and Communication, University of RouenMont-Saint-Aignan, France; ^2^Institute for Research and Innovation in Biomedicine, University of RouenMont-Saint-Aignan, France; ^3^PRIMACEN, University of RouenMont-Saint-Aignan, France; ^4^International Associated Laboratory Samuel de Champlain, University of RouenMont-Saint-Aignan, France; ^5^Institut National de la Recherche Scientifique-Institut Armand Frappier, University of QuébecLaval, QC, Canada; ^6^Région INSERM ERI28, Laboratory of Microvascular Endothelium and Neonate Lesions, University of RouenRouen, France

**Keywords:** pituitary adenylate cyclase-activating polypeptide, cerebellum, granule cells, calcium, cytoplasmic calcium stores, calcium channels, autoradiography

## Abstract

High concentrations of pituitary adenylate cyclase-activating polypeptide (PACAP) and a high density of PACAP binding sites have been detected in the developing rat cerebellum. In particular, PACAP receptors are actively expressed in immature granule cells, where they activate both adenylyl cyclase and phospholipase C. The aim of the present study was to investigate the ability of PACAP to induce calcium mobilization in cerebellar granule neurons. Administration of PACAP-induced a transient, rapid, and monophasic rise of the cytosolic calcium concentration ([Ca^2+^]_i_), while vasoactive intestinal peptide was devoid of effect, indicating the involvement of the PAC1 receptor in the Ca^2+^ response. Preincubation of granule cells with the Ca^2+^ ATPase inhibitor, thapsigargin, or the d-myo-inositol 1,4,5-trisphosphate (IP_3_) receptor antagonist, 2-aminoethoxydiphenyl borate, markedly reduced the stimulatory effect of PACAP on [Ca^2+^]_i_. Furthermore, addition of the calcium chelator, EGTA, or exposure of cells to the non-selective Ca^2+^ channel blocker, NiCl_2_, significantly attenuated the PACAP-evoked [Ca^2+^]_i_ increase. Preincubation of granule neurons with the N-type Ca^2+^ channel blocker, ω-conotoxin GVIA, decreased the PACAP-induced [Ca^2+^]_i_ response, whereas the L-type Ca^2+^ channel blocker, nifedipine, and the P- and Q-type Ca^2+^ channel blocker, ω-conotoxin MVIIC, had no effect. Altogether, these findings indicate that PACAP, acting through PAC1 receptors, provokes an increase in [Ca^2+^]_i_ in granule neurons, which is mediated by both mobilization of calcium from IP_3_-sensitive intracellular stores and activation of N-type Ca^2+^ channel. Some of the activities of PACAP on proliferation, survival, migration, and differentiation of cerebellar granule cells could thus be mediated, at least in part, through these intracellular and/or extracellular calcium fluxes.

## Introduction

Pituitary adenylate cyclase-activating polypeptide (PACAP) is a 38- or 27-amino acid peptide that was isolated from hypothalamic extracts for its ability to stimulate cAMP formation in anterior pituitary cells (Miyata et al., 1989). PACAP belongs to a superfamily of peptides that originate from a common ancestral sequence and have evolved through exon and gene duplications. In particular, PACAP27 exhibits 68% identity with vasoactive intestinal peptide (VIP). The sequence of PACAP has been very well conserved during evolution, suggesting that it may exert vital physiological activities (Vaudry et al., 2009). Indeed, numerous studies have shown that PACAP is widely expressed throughout the body and regulates a large array of biological functions, both in the central nervous system and in peripheral organs (Reglodi et al., 2012).

Three PACAP/VIP receptors, which belong to the class B family of seven-transmembrane G protein-coupled receptors, have been cloned and termed respectively PAC1, VPAC1, and VPAC2, according to their relative affinity for PACAP and VIP (Harmar et al., 2012). PAC1 recognizes PACAP27 and PACAP38 with 1000-fold higher affinity than VIP while, VPAC1 and VPAC2 exhibit similar affinity for both PACAP and VIP (Cauvin et al., 1990). All three receptors can activate a variety of second messengers including cAMP, cGMP, IP_3_, or calcium, depending on the receptor types, splice variants, G proteins, and other intracellular components expressed by each cell type (Vaudry et al., 2009). This cellular context plays a crucial role in determining the effects of PACAP, as differential expression of PAC1 splice variants is sufficient to trigger opposite activities on neuronal precursor proliferation (Nicot and DiCicco-Bloom, 2001).

It was initially shown that PACAP can increase calcium ion concentration in pituitary gonadotrope, somatotrope, and somatolactotrope cells (Canny et al., 1992; Gracia-Navarro et al., 1992; Matsuda et al., 2008). PACAP also induces calcium mobilization in chromaffin cells (Watanabe et al., 1992), hippocampal neurons (Tatsuno et al., 1992), folliculo-stellate cells (Yada et al., 1993), and type-2 astrocytes (Tatsuno and Arimura, 1994). Several transduction pathways are clearly involved in the effect of PACAP on calcium fluxes. For instance, in somatotrope cells, PACAP activates calcium mobilization in a cAMP- and protein kinase-A-dependent manner (Rawlings et al., 1993) while in gonadotrope cells, PACAP increases calcium through an inositol trisphosphate-dependent mechanism (Rawlings et al., 1994). Calcium mobilization induced by PACAP is essential for stimulation of acetylcholine (Masuo et al., 1993), catecholamine (Isobe et al., 1993), and insulin (Yada et al., 1994) release. Besides triggering neurosecretion, calcium influx also activates a variety of transcription factors that control gene expression involved in long-lasting changes of neuronal function. For instance, cAMP-dependent calcium mobilization is required for the ability of PACAP to regulate astrocyte differentiation (Cebolla et al., 2008).

In suprachiasmatic neurons, PACAP activates L-type calcium channel conductance by activating the MAPK pathway (Dziema and Obrietan, 2002). In this model, PACAP can either induce transient calcium mobilization (Dziema and Obrietan, 2002) or increase the amplitude and/or frequency of spontaneous calcium spikes (Michel et al., 2006). In cultured granule cells, some studies report that PACAP fails to increase calcium levels (Aoyagi and Takahashi, 2001) while others indicate that PACAP induces calcium mobilization (Mei, 1999). In cerebellar tissue slices, PACAP has no effect on the frequency of calcium transients but decreases the amplitude of calcium oscillations in granule cells (Cameron et al., 2007). Since PACAP exerts important neurodevelopmental and neuroprotective activities in the cerebellum (Botia et al., 2007), and since calcium plays a pivotal role in the control of cell proliferation, migration, and differentiation of granule cells (Sato et al., 2005), the aim of the present study was to investigate the ability of PACAP to induce calcium mobilization in cerebellar granule neurons and to characterize the channels involved.

## Materials and Methods

### Animals

Wistar rats were obtained from a colony raised at the University of Rouen in an accredited animal facility (approval B.76-451-04), according to the French recommendations for the care and use of laboratory animals. All experiments were performed in accordance with the French Ministry of Agriculture and the European Communities Council Directive 2010/63/UE of September 22, 2010 (approval number N/01-12-11/24/12-14) under the supervision of authorized investigators (David Vaudry, Magali Basille-Dugay). Animals were housed in a temperature-controlled room (22 ± 1°C) under a 12-h light/dark cycle and with *ad libitum* access to food and water. Pups, of both sexes, were killed by decapitation at postnatal day 8 (P8) and the cerebella were quickly dissected out.

### Reagents

The 38-amino acid form of PACAP (PACAP38) was synthesized by solid phase methodology as previously described (Jolivel et al., 2009). PACAP27 and VIP were obtained from PolyPeptide Laboratories (Strasbourg, France). Fluo-3 acetoxymethylester (Fluo-3/AM) was from Molecular Probes (Invitrogen, Cergy Pontoise, France). Thapsigargin, ethylene glycol-bis(β-aminoethylether)-N,N,N′,N′-tetraacetic acid (EGTA), nifedipine, ω-conotoxin GVIA, and ω-conotoxin MVIIC were purchased from Sigma-Aldrich (Saint-Quentin Fallavier, France). 2-aminoethoxydiphenyl borate (2APB) was from Fisher Scientific (Illkirch, France).

### Cell culture

Granule cell suspensions were prepared from cerebella of P8 rats, as previously described (Kaddour et al., 2013). For calcium experiments, dispersed cells were seeded on poly-l-lysine-coated glass coverslips in Falcon 3001 dishes at a density of 1 × 10^6^ cells/mL in a cultured medium consisting of 75% DMEM and 25% Ham’s F12 supplemented with 10% fetal bovine serum, 25 mM KCl, and 1% antibiotic antimycotic solution. Cells were grown at 37°C in a humidified incubator with an atmosphere of 5% CO_2_/95% air. After 20 h, cytosine arabinoside, a mitotic inhibitor, was added at a final concentration of 5 μM to avoid replication of non-neuronal cells. In these conditions, the culture mainly contains cerebellar granule cells and contaminant cells are mostly astrocytes, which can be recognized based on their morphology (Levi et al., 1989).

### Calcium measurement

Forty-eight hours after plating, granule cells were loaded at 37°C for 45 min with 5 μM fluo-3/AM diluted in culture medium. Then, the calcium-dye-probe was washed off and replaced with a Ringer’s solution containing 10 mM Hepes, 120 mM NaCl, 2 mM KCl, 1.8 mM CaCl_2_, 1 mM MgCl_2_, and 6 mM glucose (pH 7.4). Under certain conditions, pharmacological agents were added in the Ringer’s solution 30 min before mounting the glass coverslip on the stage of a Nikon inverted microscope (Eclipse TE200) equipped with a Nikon 60x, 1.4 NA, oil-immersion objective. Fluo-3/AM was excited with the 488-nm laser line, and the emitted fluorescence was recorded with a 500-nm long-pass filter on a Noran OZ confocal laser-scanning microscope (Noran Instruments, Middleton, WI, USA), equipped with a standard argon/kripton laser for illumination. Images were acquired as a time series of scan of the same focal plane (512 × 480 pixels at 7.5 images/s) and data processing was carried out using the Intervision software (Noran Instruments). The cells were continuously perifused with Ringer’s solution at constant flow rate (2 mL/min) and temperature (37°C). The perifusion system was also used to deliver test substances at the vicinity of the cultured neuroblasts. Any modification of fluorescence directly reflects a change of intracellular calcium concentration ([Ca^2+^]_i_). To determine the increase of [Ca^2+^]_i_, the value of the peak amplitude as well as the area under the curve (AUC) of each response profile were calculated by using the PRISM Software (GraphPad Software, San Diego, CA, USA). Results are expressed as histograms showing (i) the distribution of cells according to the amplitude of their response to PACAP and (ii) the mean AUCs of their [Ca^2+^]_i_ increase.

### Binding studies

PACAP27 was radioiodinated by means of the lactoperoxidase technique as previously described (Basille et al., 1994). The radioligand was purified by reverse-phase HPLC on a Vydac C_18_ column (25 × 0.46 cm; Sigma-Aldrich), using a gradient of acetonitrile/water containing 0.1% TFA. The specific radioactivity of the tracer was approximately 800 Ci/mmol.

Twenty-μm-thick sections or granule cells from P8 rat cerebella cultured for 1 day were preincubated at 20°C in 50 mM Tris buffer (pH 7.4) supplemented with 1% bovine serum albumin (BSA), 32 mM saccharose, 5 mM MgCl_2_, and 0.5 μg/mL bacitracin, for 30 or 10 min, respectively. Sections or cells were incubated with [^125^I]PACAP27 (40 or 400 pM, respectively) at 20°C for 1 h in the same buffer, supplemented with 2% BSA. To visualize non-specific binding, slices, or cells were incubated with the radioligand in the presence of 10^−6^ M PACAP38. At the end of the incubation, sections, or cells were washed with cold Tris buffer and dried under an air stream. Finally, slices were apposed onto Hyperfilm-3H (GE Healthcare, Les Ulis, France) for 6 days and the radioactivity associated with the cells was counted in a gamma-counter (LKB, Wallac, Rockville, MI, USA). Tissue slices were photographed by means of a computer-assisted image-analysis station (Samba, Grenoble, Lyon).

### Statistical analysis

Data are expressed as mean ± SEM values from at least three independent experiments. Statistical analyses were conducted with the PRISM software.

## Results

### Effect of PACAP38, PACAP27, and VIP on [Ca^2+^]i

**Figure 1:**
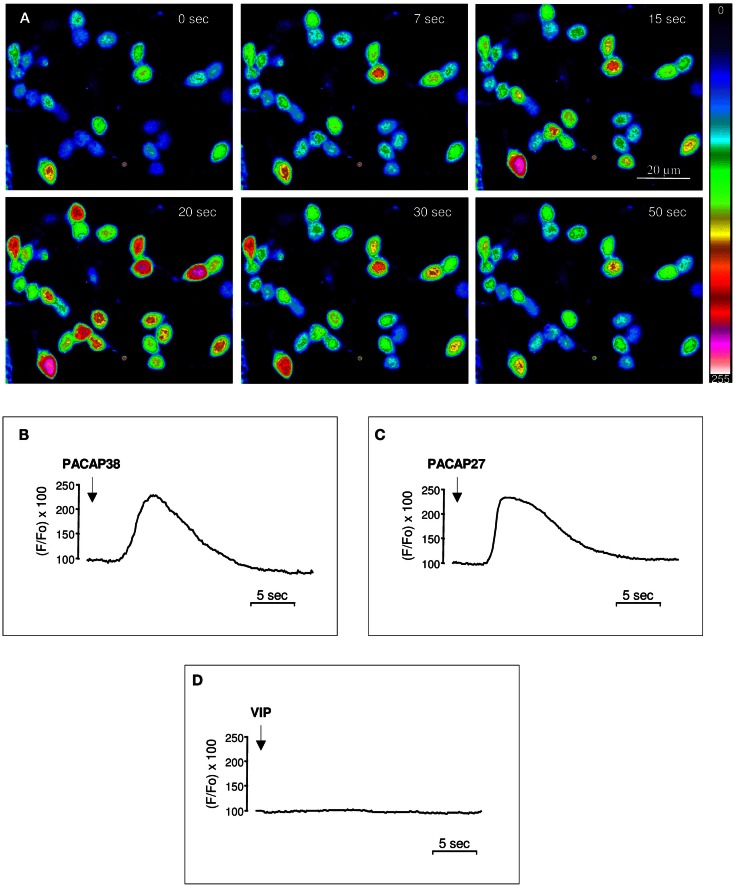
**Effect of PACAP38, PACAP27, or VIP on [Ca^2+^]_i_ in cultured cerebellar granule cells**. **(A)** Time series of pseudocolor images illustrating the [Ca^2+^]_i_ changes in cultured granule cells loaded with the Ca^2+^-sensitive dye fluo-3/AM after infusion of 10^−6^ M PACAP38 at the vicinity of the cells. The pseudocolor scale indicates the corresponding [Ca^2+^]_i_ changes expressed in arbitrary units. **(B–D)** Typical profiles illustrating the time-course of the variation of the fluorescence ratio evoked by a single application of 10^−6^ M PACAP38 **(B)**, PACAP27 **(C)**, or VIP **(D)** on [Ca^2+^]_i_ in cultured granule cells. The arrows indicate the onset of peptide application.

Perifusion of cultured cerebellar granule cells with 10^−6^ M PACAP38 provoked a marked increase of [Ca^2+^]_i_ in more than 90% of the cells (Figure 1A). In all responding cells [Ca^2+^]_i_ increased rapidly, i.e., within less than 5 s following PACAP infusion, and reached a maximum after 15–20 s, but the amplitude of the response was variable depending on the cell (Figure 1A). The response profile determined as the ratio between the fluo-3 fluorescence intensity under resting conditions and after exposure to test substances, revealed that both PACAP38 and PACAP27 (10^−6^ M) induced a transient, rapid, and monophasic increase in [Ca^2+^]_i_ followed by gradual return to baseline (Figures 1B,C) while application of vehicle had no effect. Conversely, administration of 10^−6^ M VIP did not induce any modification of the [Ca^2+^]_i_ level (Figure 1D). When the same cells were exposed to a second pulse of PACAP, the stimulatory effect of the peptide on [Ca^2+^]_i_ was totally abolished (data not shown).

### Contribution of intracellular Ca^2+^ pools in the PACAP38-evoked increase of [Ca^2+^]i

**Figure 2:**
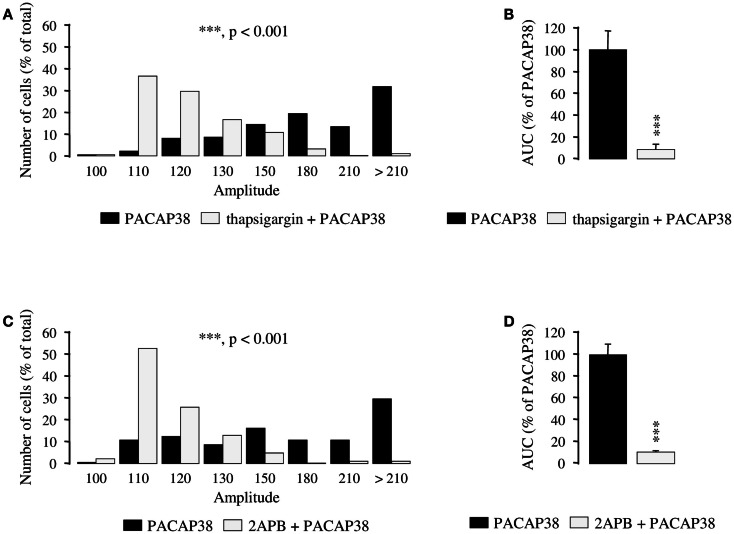
**Involvement of intracellular calcium stores in the mechanism of action of PACAP38 in cerebellar granule cells**. **(A–D)** Effect of a single application of 10^−6^ M PACAP38 on the amplitude **(A,C)** and the area under the curve **(B,D)** of the [Ca^2+^]_i_ response in the absence (black bars) or presence (white bars) of 10^−6^ M thapsigargin, a Ca^2+^ ATPase inhibitor, or 10^−5^ M 2APB, a membrane permeable d-myo-inositol 1,4,5-trisphosphate receptor antagonist. Thapsigargin and 2APB were added 15 and 30 min before application of the pulse of PACAP, respectively. **(A,C)** Histograms showing the distribution of granule cells according to the amplitude of their response to PACAP. Each graph represents the analysis of a least 50 cells from 3 different cultures. 2APB, 2-aminoethoxydiphenyl borate; AUC, area under the curve. ***p < 0.001.

Preincubation of granule cells with the Ca^2+^ ATPase inhibitor thapsigargin (10^−6^ M; 15 min) induced a substantial reduction of the amplitude of the [Ca^2+^]_i_ response to 10^−6^ M PACAP38 in most cells (*p* < 0.001; Figure 2A). Indeed, in the absence of thapsigargin, more than 80% of the responding cells exhibited at least a 50% increase of their fluorescence intensity whereas in the presence of the inhibitor, 80% of the cells exhibited an increase of their fluorescence intensity that was lower than 30% (Figure 2A). As shown in Figure 2B, thapsigargin reduced by 86% the mean AUC of the PACAP38-evoked [Ca^2+^]_i_ response (*p* < 0.001). Preincubation of granule cells with the permeable d-myo-inositol 1,4,5-trisphosphate receptor antagonist 2APB (10^−5^ M) markedly reduced the [Ca^2+^]_i_ response to 10^−6^ M PACAP38. In the presence of 2APB, more than 90% of responding cells exhibited an increase of their fluorescence intensity that was lower than 30% (*p* < 0.001; Figure 2C). As shown in Figure 2D, 2APB reduced by 89% the mean AUC of the PACAP38-evoked [Ca^2+^]_i_ response (*p* < 0.001).

### Contribution of extracellular Ca^2+^ in the PACAP38-evoked stimulation of [Ca^2+^]i

**Figure 3:**
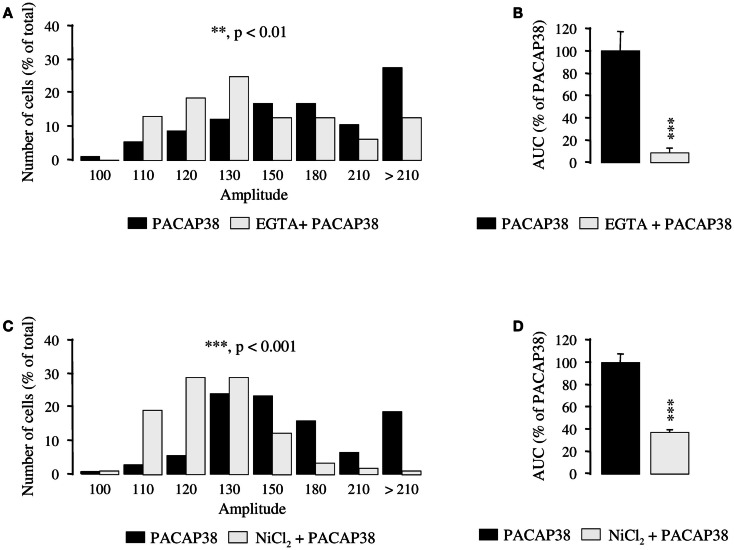
**Involvement of calcium influx in the mechanism of action of PACAP38 in cerebellar granule cells**. **(A–D)** Effect of a single application of 10^−6^ M PACAP38 on the amplitude **(A,C)** and the area under the curve **(B,D)** of the [Ca^2+^]_i_ response in the absence (black bars) or presence (white bars) of 6 mM EGTA, a Ca^2+^ chelator, or 3 mM NiCl_2_, a blocker of voltage-operated calcium channels. EGTA and NiCl_2_ were added to the incubation medium 10 min before application of the pulse of PACAP. **(A,C)** Histograms showing the distribution of granule cells according to the amplitude of their response to PACAP. Each graph represents the analysis of a least 50 cells from 3 different cultures. AUC, area under the curve; EGTA, ethylene glycol-bis(β-aminoethylether)-N,N,N′,N′-tetraacetic acid; NiCl_2_, nickel chloride. **p < 0.01, ***p < 0.001.

Preincubation of granule cells with the calcium chelator EGTA (6 mM; 10 min), significantly attenuated the amplitude of the [Ca^2+^]_i_ response to 10^−6^ M PACAP38 (*p* < 0.01; Figure 3A) and diminished by 91% the AUC of the PACAP38-evoked [Ca^2+^]_i_ increase (*p* < 0.001; Figure 3B). Similarly, a 10-min preincubation with 3 mM NiCl_2_, a blocker of voltage-operated calcium channels (VOCCs), significantly reduced the amplitude of the [Ca^2+^]_i_ peak in response to 10^−6^ M PACAP38 (*p* < 0.001; Figure 3C). Indeed, while 90% of the responding cells had a ratio of fluorescence intensity greater than 130 in the absence of NiCl_2_, 79% of them had an amplitude lower than 130 in the presence of the VOCC blocker (Figure 3C). Furthermore, addition of NiCl_2_ to the Ringer’s solution reduced by 62% the AUC of the PACAP38-evoked [Ca^2+^]_i_ response (*p* < 0.001; Figure 3D).

### Absence of effect of EGTA on [^125^I]PACAP27 binding

**Figure 4:**
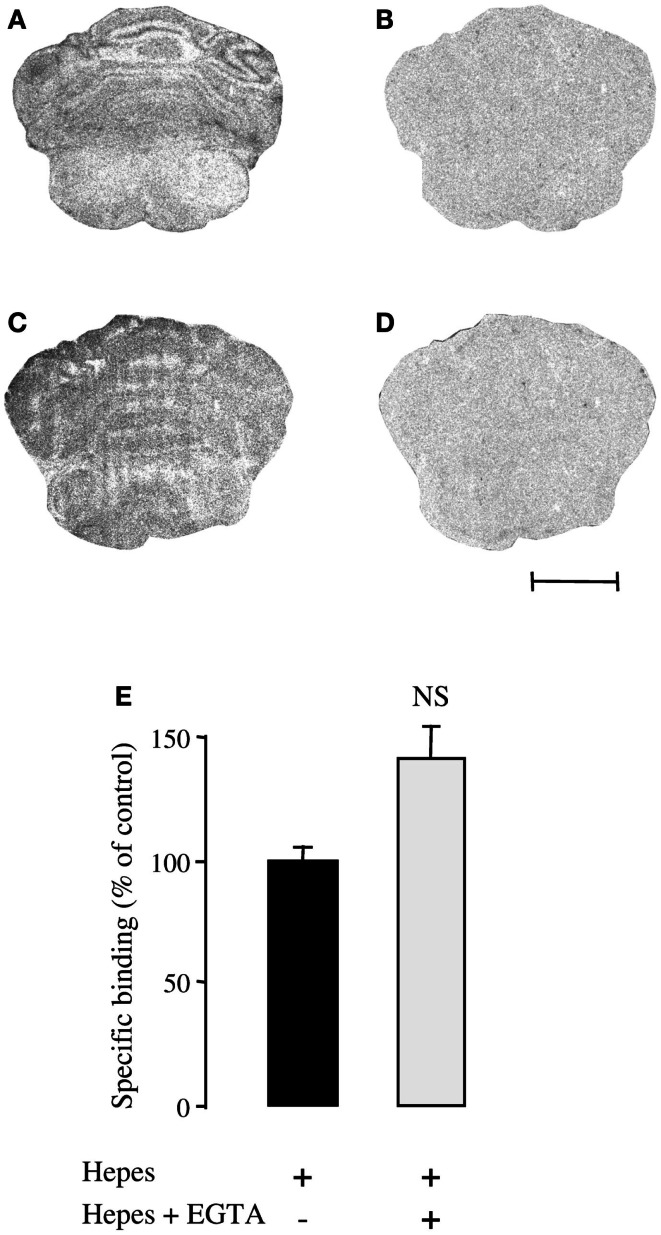
**Effect of the Ca^2+^ chelator EGTA (6 mM) on [^125^I]PACAP27 binding to cerebellar tissue slices or cerebellar granule cells**. **(A–D)** Autoradiographic visualization of [^125^I]PACAP27 binding sites in the absence **(A,C)** or presence of 10^−6^ M PACAP38 **(B,D)** in consecutive sections of P8 rat cerebellum. Scale bar = 1.7 mm. **(E)** Histograms showing specific binding of [^125^I]PACAP27 in the absence (black bars) or presence (white bars) of 6 mM EGTA on cultured cerebellar granule cells. EGTA, ethylene glycol-bis(β-aminoethylether)-N,N,N′,N′-tetraacetic acid. NS, not significantly different from Hepes buffer.

To verify that EGTA did not influence PACAP binding on its recognition sites, autoradiography experiments were carried out using [^125^I]PACAP27 as a radioligand. As previously reported [^125^I]PACAP27 binding was observed in the external granule cell layer of P8 rat cerebella (Figure 4A). Addition of 10^−6^ M PACAP38 to the incubation medium completely displaced [^125^I]PACAP27 binding (Figure 4B). In the presence of 6 mM EGTA, specific binding of [^125^I]PACAP27 on cerebellar tissue slices was not impaired (Figure 4C) and displacement of [^125^I]PACAP27 binding by PACAP38 was not affected (Figure 4D). Binding experiments were also performed on 1-day-old cultured granule cells with [^125^I]PACAP27 as a radioligand and it appeared that 6 mM EGTA did not modify the specific binding of [^125^I]PACAP27 on cultured cells (Figure 4E).

### Involvement of N-type calcium channels in the PACAP38-evoked stimulation of [Ca^2+^]i

**Figure 5:**
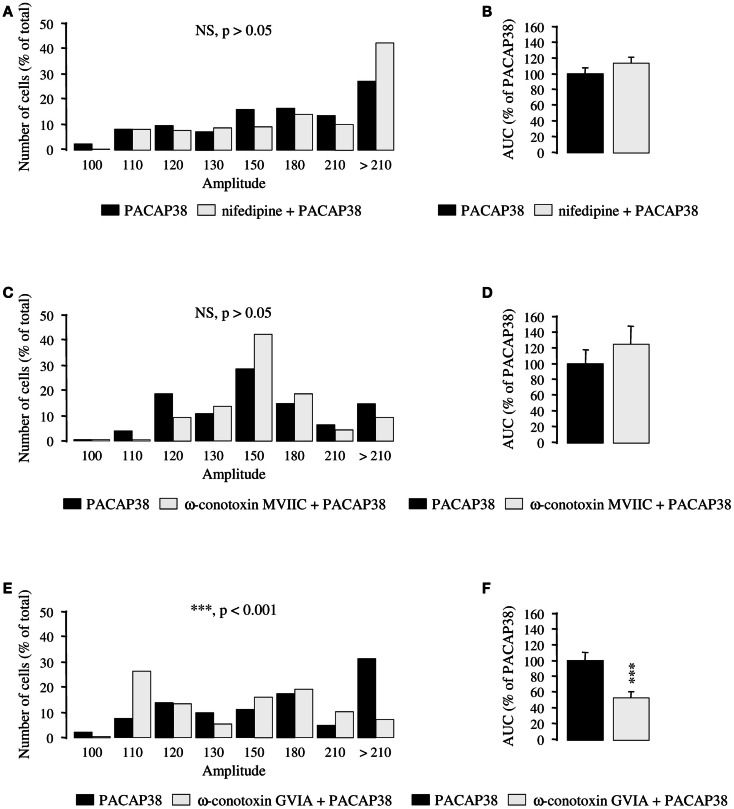
**Effect of selective blockers of voltage-operated calcium channels on calcium influx induced by PACAP38 in cerebellar granule cells**. **(A–F)** Histograms showing the effect of a single application of 10^−6^ M PACAP38 on the amplitude **(A,C,E)** and the area under the curve **(B,D,F)** of the [Ca^2+^]_i_ response in the absence (black bars) or presence (white bars) of 10^−5^ M of the L-type Ca^2+^ channel blocker nifedipine **(A,B)**, 10^−6^ M of the P- and Q-type Ca^2+^ channel blocker ω-conotoxin MVIIC **(C,D)**, or 10^−6^ M of the N-type Ca^2+^ channel blocker ω-conotoxin GVIA **(E,F)**. Nifedipine, ω-conotoxin MVIIC, and ω-conotoxin GVIA were added to the incubation medium 30 min before application of the pulse of PACAP38. **(A,C,E)** Histograms showing the distribution of granule cells according to the amplitude of their response to PACAP. Each graph represents the analysis of a least 50 cells from 3 different cultures. AUC, area under the curve; NS, not significantly different from the PACAP38; ***p < 0.001.

To determine which type of Ca^2+^ channel is responsible for the stimulatory effect of PACAP38 on calcium influx, selective blockers of VOCCs were used. A 30-min incubation of cultured granule cells with the L-type Ca^2+^ channel blocker nifedipine (10^−5^ M; Figures 5A,B) or the P- and Q-type Ca^2+^ channel blocker ω-conotoxin MVIIC (10^−6^ M; Figures 5C,D) did not significantly modify the amplitude of the [Ca^2+^]_i_ response to 10^−6^ M PACAP38 (*p* > 0.05; Figures 5A,C). Consistent with these observations, nifedipine, and ω-conotoxin MVIIC did not impair the AUCs of the PACAP38-induced [Ca^2+^]_i_ rise (*p* > 0.05; Figures 5B,D). In contrast, preincubation of cells with the N-type Ca^2+^ channel blocker ω-conotoxin GVIA (10^−6^ M; 30 min; Figures 5E,F) provoked a significant decrease of the amplitude of the [Ca^2+^]_i_ increase induced by 10^−6^ M PACAP38, with 76 and 52% of responding cells exhibiting a ratio of fluorescence intensity greater than 130 in the absence or presence of ω-conotoxin GVIA, respectively (*p* < 0.001; Figure 5E). Furthermore, ω-conotoxin GVIA reduced by 46% the AUC of the PACAP38-evoked [Ca^2+^]_i_ response (*p* < 0.001; Figure 5F).

## Discussion

The respective contribution of extracellular and intracellular Ca^2+^ pools in PACAP-induced [Ca^2+^]_i_ increase in cerebellar neuroblasts has not been previously investigated. Because primary cultures of rat cerebellar granule cells are mainly composed of a single population of cells, the effect of PACAP on the [Ca^2+^]_i_ response could be monitored on a large number of neurons. Thus, we found that, in 90% of granule cells, infusion of 10^−6^ M PACAP38 or PACAP27 induced a transient, rapid, and monophasic [Ca^2+^]_i_ rise, similar to the PACAP-induced Ca^2+^ response observed in neural NG108-15 cells (Holighaus et al., 2011) but different from that observed in rat pancreatic acinar AR42J cells (Barnhart et al., 1997) or human neuroblastoma NB-OK-1 cells (Delporte et al., 1993) in which the initial peak is followed by a plateau phase that lasts for approximately 2–3 min before the return of [Ca^2+^]_i_ to baseline level. These differences could be explained by the diversity of the PACAP receptor subtype repertoire expressed in each cell type and by the different intracellular signaling systems involved. For instance, in rat gonadotrophs, PACAP stimulates Ca^2+^ oscillations through activation of VPAC1 coupled to a PTX-insensitive G protein and phospholipase C-β (Hezareh et al., 1996) whereas, in cerebellar neurons, in which VIP failed to affect [Ca^2+^]_i_, the ability of PACAP to induce [Ca^2+^]_i_ rise can be ascribed to activation of the PAC1 receptor, as previously reported in primary cultures of rat cortical neurons and astrocytes (Grimaldi and Cavallaro, 1999). In neural NG108-15 cells, the PACAP38-evoked [Ca^2+^]_i_ response is mainly mediated by the PAC1 hop1 splice variant (Mustafa et al., 2007; Holighaus et al., 2011), an isoform also expressed in granule neurons (Kienlen-Campard et al., 1997). Interestingly, this hop isoform has been reported to be mandatory for calcium influx in cortical precursors (Yan et al., 2013). In β-islet cells, the PAC1 TM4 splice variant increases [Ca^2+^]_i_ by stimulating Ca^2+^ influx *via* a L-type Ca^2+^ channel without activating adenylyl cyclase or phospholipase C in response to PACAP (Chatterjee et al., 1996). In cerebellar granule cells, the TM4 splice variant is expressed at a very low level (Chatterjee et al., 1996) and is thus probably not functionally relevant considering the strong activation of the cAMP pathway evoked by PACAP (Basille et al., 1993, 1995).

It has previously been demonstrated that, in cerebellar granule neurons, PACAP activates the phospholipase C pathway (Basille et al., 1995). We have thus explored the possible contribution of intracellular Ca^2+^ stores in the stimulatory effect of PACAP on [Ca^2+^]_i_. The results showed that depletion of the endoplasmic reticulum Ca^2+^ store with the Ca^2+^ ATPase inhibitor thapsigargin reduced the amplitude and the AUC of the PACAP-evoked [Ca^2+^]_i_ response in cerebellar neurons. Consistent with the notion that intracellular Ca^2+^ from IP_3_-sensitive Ca^2+^ pools could play an important role in the mechanism of action of PACAP, incubation of the cells with 2APB, a cell permeable d-myo-inositol 1,4,5-trisphosphate receptor antagonist, decreased the [Ca^2+^]_i_ response to PACAP. Such a contribution of intracellular Ca^2+^ stores to the PACAP-evoked [Ca^2+^]_i_ response has already been reported in rat gonadotrophs (Rawlings et al., 1994), in rat acinar AR42J cells (Barnhart et al., 1997), and in human neutrophils (Harfi and Sariban, 2006).

Alongside, suppression of extracellular Ca^2+^ by EGTA or exposure of cells to the non-selective Ca^2+^ channel blocker NiCl_2_, also attenuated the stimulatory effect of PACAP38 on [Ca^2+^]_i_, indicating that Ca^2+^ influx is also required for the transient phase of the [Ca^2+^]_i_ increase. Recruitment of both intracellular and extracellular sources of Ca^2+^ after activation of PAC1 receptors has already been reported in several models including the rat acinar cell line AR42J (Barnhart et al., 1997), the human neuroblastoma cell line NB-OK-1 (Delporte et al., 1993), and primary cultures of rat cortical neurons (Grimaldi and Cavallaro, 1999). To determine which type of Ca^2+^ channel was responsible for the stimulatory effect of PACAP on calcium influx, selective blockers of VOCCs were used. Preincubation of granule cells with the N-type Ca^2+^ channel blocker ω-conotoxin GVIA decreased the PACAP-evoked [Ca^2+^]_i_ response, whereas the L-type Ca^2+^ channel blocker nifedipine and the P- and Q-type Ca^2+^ channel blocker ω-conotoxin MVIIC had no effect. These findings contrast with previous data showing the involvement of L-type Ca^2+^ channels in the stimulatory effect of PACAP on human neutrophils (Harfi et al., 2005), on porcine somatotrope cells (Martinez-Fuentes et al., 1998) or on bovine adrenal chromaffin cells (Tanaka et al., 1996) and the implication of T-type Ca^2+^ channels in mouse adrenal chromaffin cells (Hill et al., 2011). Nevertheless, other factors acting on cerebellar granule neurons, such as IGF-1, have already been shown to regulate N-type Ca^2+^-channels (Blair and Marshall, 1997).

Ca^2+^ is an essential intracellular messenger required for numerous cellular functions (Carafoli et al., 2001). For instance, during development, modifications of intracellular Ca^2+^ concentrations have been shown to regulate proliferation (Owens et al., 2000), migration (Komuro and Rakic, 1992), differentiation (Benquet et al., 2002; Ronn et al., 2002), and apoptosis (Turner et al., 2002) of immature neurons. Various studies suggest that, depending on the pool involved, Ca^2+^ can exert different effects on immature neurons. For instance, in rat cortical neurons, Ca^2+^ release from intracellular stores is implicated in neurite elongation, while Ca^2+^ influx regulates dendritic branching (Ramakers et al., 2000). In the immature cerebellum, a transient elevation of intracellular Ca^2+^ levels increases the migration rate of granule neuroblasts (Komuro and Rakic, 1996) and the thapsigargin-sensitive Ca^2+^ store plays an essential role in growth and maturation of cerebellar granule cells (Yao et al., 1999). Even though the implication of Ca^2+^ on granule cell development is now well established, the neurotrophic factors able to control Ca^2+^ levels in cerebellar neuroblasts are not yet clearly identified. Nevertheless, peptides acting on G protein-coupled receptors are thought to be important mediators to control neurite outgrowth and growth cone guidance in cerebellar granule cells through a Ca^2+^-dependent pathway (Xiang et al., 2002).

Granule cells are, by far, the major population of interneurons in the cerebellum and they represent the main source of glutamate, the second most abundant population being GABA-ergic neurons (Voogd and Glickstein, 1998). Previous studies have demonstrated that plasticity of granule cells can be modulated by neuropeptides (Cote et al., 1999; Yacubova and Komuro, 2002). In particular, in cerebellar granule cells, PACAP has been shown to inhibit proliferation (Nicot et al., 2002), stop migration (Cameron et al., 2007), protect from apoptosis (Vaudry et al., 2000), and promote differentiation (Gonzalez et al., 1997). *In vivo*, PACAP receptors are expressed by precursors of cerebellar granule cells in the external granule cell layer (Basille et al., 1993) and PACAP administration increases the number of mature neurons in the post-migratory internal granule cell layer (Vaudry et al., 1999). The implication of adenylyl cyclase and MAP kinases in the effects of PACAP have been extensively investigated (Villalba et al., 1997; Vaudry et al., 1998; Nicot et al., 2002) but the contribution of PACAP-induced Ca^2+^ increase in maturation of cerebellar neuroblasts is still poorly understood. So far, it has only been shown that calcium is involved in the inhibitory effect of PACAP on granule cell migration (Cameron et al., 2007). However, it is well established that potassium depolarization-induced Ca^2+^ entry is essential for granule cell survival (Gallo et al., 1987), suggesting that the antiapoptotic effect of PACAP may involve calcium mobilization. As reported with cortical neurons, PACAP may induce BDNF expression in a Ca^2+^-dependent manner to indirectly promote granule cell survival (Shintani et al., 2005; Kokubo et al., 2009). The stimulation of granule cell proliferation by calcium influx is suppressed by nifedipine but not by ω-conotoxin GVIA (Borodinsky and Fiszman, 1998), suggesting that the ability of PACAP to block granule cell division does not depend on calcium regulation. Consistent with this latter hypothesis, it has been shown that, in cortical neurons, PACAP inhibits cell proliferation through activation of the cAMP signaling pathway (Nicot and DiCicco-Bloom, 2001). Finally, potassium depolarization and NMDA treatment require calcium influx to induce neurofilament assembly in cerebellar granule cells (Bui et al., 2003), supporting the idea that PACAP-induced neurite outgrowth also requires calcium mobilization.

Altogether, these data indicate that some of the activities of PACAP on cerebellar granule cell proliferation, survival, migration, and differentiation involve, at least in part, intracellular and/or extracellular calcium mobilization, but further investigations are needed to decipher the precise role of calcium in each process.

## Conflict of Interest Statement

The authors declare that the research was conducted in the absence of any commercial or financial relationships that could be construed as a potential conflict of interest.

## Abbreviations

2APB, 2-aminoethoxydiphenyl borate; Ca^2+^, calcium; [Ca^2+^]_i_, intracellular calcium concentration; EGTA, ethylene glycol-bis(β-aminoethylether)-N,N,N′,N′-tetraacetic acid; P, postnatal day; PACAP, pituitary adenylate cyclase-activating polypeptide; PACAP27, 27-amino acid form of PACAP; PACAP38, 38-amino acid form of PACAP; VIP, vasoactive intestinal peptide.
